# Egg Intake Is Associated with Lower Risks of Impaired Fasting Glucose and High Blood Pressure in Framingham Offspring Study Adults

**DOI:** 10.3390/nu15030507

**Published:** 2023-01-18

**Authors:** Melanie M. Mott, Xinyi Zhou, M. Loring Bradlee, Martha R. Singer, Ioanna Yiannakou, Lynn L. Moore

**Affiliations:** 1 Department of Medicine, Preventive Medicine and Epidemiology, Boston University Chobanian & Avedisian School of Medicine, 72 East Concord St, Boston, MA 02118, USA; 2 DynaMed at EBSCO Information Services, 10 Estes Street, Ipswich, MA 01938, USA

**Keywords:** eggs, fasting glucose, blood pressure, diet patterns, diabetes, high blood pressure, prospective study

## Abstract

The association between egg consumption and cardiometabolic risk factors such as high blood pressure (HBP) and impaired fasting glucose (IFG) or type 2 diabetes (T2D) is still under debate. This study examines the association between egg consumption and these outcomes among 2349 30–64 year-old adults in the prospective Framingham Offspring Study. Diet was assessed using three-day dietary records. Potential confounders retained in the final models included age, sex, body mass index, and other dietary factors. The analysis of covariance and Cox proportional hazard’s models were used to assess the relevant continuous (i.e., FG, SBP, DBP) and categorical (i.e., T2D, HBP) outcomes. Consuming ≥5 eggs per week was associated with lower mean FG (*p* = 0.0004) and SBP (*p* = 0.0284) after four years of follow-up. Higher egg intakes led to lower risks of developing IFG or T2D (HR: 0.72; 95% CI: 0.51–1.03) and high blood pressure (HBP) (HR: 0.68; 0.50–0.93). The beneficial effects of egg consumption were stronger in combination with other healthy dietary patterns. This study found that regular egg consumption as part of a healthy diet had long-term beneficial effects on blood pressure and glucose metabolism and lowered the long-term risks of high blood pressure and diabetes.

## 1. Introduction

Cardiovascular disease (CVD) is the leading cause of death in the United States (U.S.) [1]. For many years, it has been suggested that eggs may increase CVD risk through their dietary cholesterol content, but in recent years, this belief has been increasingly questioned [2,3] In 2015, the U.S. Dietary Guidelines were revised to say that dietary cholesterol was no longer considered a nutrient of concern for overconsumption [4]. However, since that time, an accumulating body of conflicting evidence has continued to grow [5,6,7,8].

Eggs are a key source of dietary cholesterol and may be an important source of protein in the U.S. diet, but their possible role in the evolution of cardiometabolic risk is still not well understood. Two key risk factors for the development of CVD include impaired fasting glucose (IFG) and high blood pressure (HBP) [9]. One meta-analysis found egg intake to be positively associated with incident T2D [10], while two others found no such association [11,12]. Some short-term randomized clinical trials also found no adverse effect of eggs on glucose metabolism in either healthy adults or individuals with prevalent T2D [13,14,15]. It has been suggested that the inconsistent findings in the literature could be due to such things as uncontrolled confounding by other dietary factors or to differences in study populations [16,17]. 

There are a number of different types of studies of egg consumption and blood pressure. For example, a cross-sectional study from the National Health and Nutrition Examination Survey found that consuming two eggs per day had no adverse association with blood pressure [18], and a meta-analysis of clinical trials also found no association [19]. Some other short-term interventions have had similar findings [10,20,21]. However, in a recent prospective cohort study, egg-derived dietary protein was found to be associated with a lower risk of high blood pressure in Chinese adults [22]. 

For many years, the U.S. Dietary Guidelines for Americans recommended restricting egg intake as a means of limiting dietary cholesterol intake, thereby lowering the risk of CVD [23]. This guidance was revised in the 2015 Dietary Guidelines when dietary cholesterol was determined not to be a nutrient of concern for overconsumption [4]. Since that time, an accumulating body of conflicting evidence has continued to grow [5,6,7,8]. 

The overall goal of this study was to examine the impact of egg consumption, alone and in the context of other eating patterns, on fasting glucose and blood pressure among adults in the Framingham Offspring Study (FOS).

## 2. Materials and Methods

### 2.1. Study Population

The Framingham Offspring Study began in 1971 with the enrollment of 5124 offspring of the original Framingham Heart Study cohort [24]. Generally, participants have been examined approximately every four years for the development of CVD and other health outcomes. At each exam visit, participants completed questionnaires and interviews, had measurements taken (e.g., anthropometric measures, blood pressure), and blood drawn. Diet was assessed using three-day diet records between the third and fifth examination cycles (1983–1995). 

**Figure 1:**
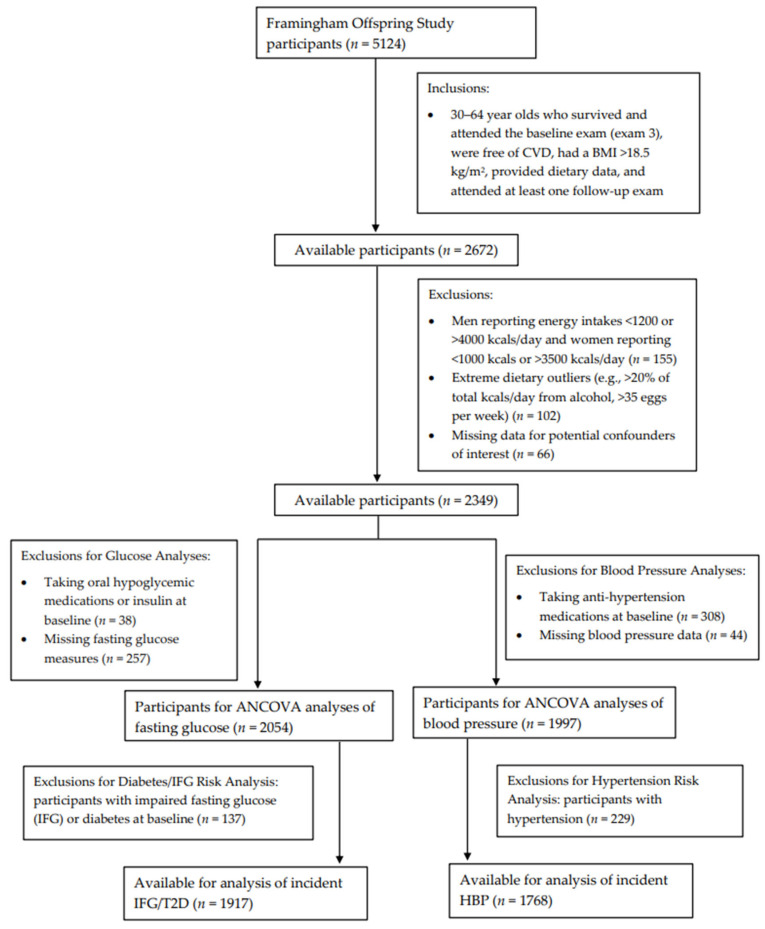
Flowchart of study participants.

Figure 1 shows the inclusion and exclusion criteria for participants included in these analyses. Specifically, we included adults, ages 30 to 64 years, who survived, attended the third examination visit, provided dietary record data, had a BMI >18.5 kg/m^2^, were free of CVD, and had at least one follow-up exam after the initial dietary assessment (*n* = 2672). Men who reported energy intakes of <1200 kcals or >4000 kcals per day and women reporting <1000 kcals or >3500 kcals per day (*n* = 155) were excluded. Those who reported consuming >20% of total energy intake per day from alcohol were also excluded, as were those consuming >35 eggs per week (*n* = 102). Those with missing data for potential confounders of interest (*n* = 66) were also excluded.

Finally, additional exclusions were made separately for fasting glucose and blood pressure analyses, which were assessed at the sixth examination visit (1995–1998). For continuous fasting glucose analyses, individuals taking oral hypoglycemic medications or insulin at baseline (*n* = 38) or with missing fasting glucose measures (*n* = 257) were excluded, leaving a sample of 2054 men and women for the assessment of egg intake on subsequent fasting glucose. Additionally, follow-up for incident IFG or T2D occurred from exam visits five through eight (1995–2008). For these analyses, individuals with prevalent IFG or diabetes (either type 1 or type 2) (*n* = 137) at baseline were also excluded, leaving 1917 participants for analyses with incident IFG or T2D. For the analysis of adjusted mean blood pressure levels, participants taking antihypertensive medications at baseline (*n* = 308) or missing blood pressure data (*n* = 44) were excluded, leaving 1997 men and women. An additional 229 individuals with prevalent HBP were excluded from analyses of incident HBP. 

### 2.2. Dietary Assessment

Three-day dietary records (two weekdays and one weekend day) were completed during the third and fifth exam cycles. Participants were instructed by a trained nutritionist in using standard protocols and two-dimensional food models in the completion of the dietary records. After the three days of dietary records were returned, a single senior nutritionist reviewed the diet record and debriefed the subjects on any questions or needed clarifications. 

Approximately 70% of participants completed these food records, resulting in approximately 16,000 days of dietary data. The dietary records were entered (by the same nutritionist who carried out the debriefing) into the Nutrient Data System (NDS) of the University of Minnesota, in accordance with standardized protocols, and mean intakes of macro- and micronutrients were derived from the NDS [25]. Researchers from Boston University linked the underlying food codes from NDS with the United States Department of Agriculture (USDA) food pyramid serving data for eggs and other food groups as previously described [26]. Total egg intake from these data were derived from whole foods (whole eggs), composite foods (e.g., mayonnaise), and mixed dishes (e.g., quiche). In the NDS, participants consuming egg whites only (rather than whole eggs) were credited with 2/3 of one egg for each egg consumed since an egg white is approximately 2/3 of a whole egg, by weight. The above linkage of dietary record data with USDA Food Pyramid serving data also allowed for the calculation of Healthy Eating Index scores as a measure of diet quality for each participant [27].

### 2.3. Main Outcome Measures

During the time period of these analyses (exams three to eight), fasting blood specimens were drawn at each examination visit following an overnight fast (12 h) for the estimation of glucose, insulin, lipid levels, and other hemostatic factors [28]. Fasting plasma glucose was measured in fresh specimens using a hexokinanse reagent kit. Among participants without prevalent T2D who had an elevated fasting glucose level (≥110 mg/dL), a 75 g oral glucose tolerance test was administered using 1997 American Diabetes Association standards to identify participants with T2D [29]. Extreme fasting glucose values for nine subjects were truncated to 200 mg/dL to eliminate the influence of these outliers. Systolic and diastolic blood pressures were measured following the standard procedure of the Joint National Committee on Prevention, Detection, Evaluation, and Treatment of High Blood Pressure [30]. Two visit-specific measurements were taken with a mercury sphygmomanometer and were used to estimate mean SBP and DBP at each visit.

For these analyses, IFG was defined as a fasting glucose of 110–125 mg/dL; this cutoff value was chosen as a level that is known to confer a higher risk for metabolic disorders and/or cardiovascular disease [31]. Incident T2D was defined as a fasting glucose ≥ 126 mg/dL or the taking of a glucose-lowering medication. Incident HBP was defined as any of the following: a mean SBP ≥ 140 mmHg or mean DBP ≥ 90 mmHg at two consecutive exams, a mean systolic blood pressure ≥ 160 mmHg or diastolic blood pressure ≥ 95 mmHg at a single exam, or the use of antihypertensive medication for the purpose of lowering blood pressure.

### 2.4. Potential Confounders

There were a number of potential confounders considered in these analyses. Only those factors that changed the adjusted mean values or hazard ratio estimates by 5% or more were included in the final models. Factors that were explored included education level (a self-reported level of education of ≤12 years vs. ≥13 years), physical activity index, cigarette smoking (current smoking status and number of cigarettes smoked per day), implausible dietary intake, total energy intake, dietary sodium intake, dietary potassium intake, Healthy Eating Index scores, and other dietary factors including total fruits, vegetables and dietary fiber consumed. 

For the fasting glucose and blood pressure analyses, age, sex, dietary fiber, and baseline body mass index (BMI) were included in the final models. For the analyses examining the risk of incident T2D or HBP,, age, sex, baseline BMI, and the consumption of solid fats/alcoholic beverages/added sugars (SoFAAs) were included in the final models. 

Height and weight were measured with the shoes off and with the subject wearing a hospital gown. A standard beam balance scale with a stadiometer was used to take duplicate measures at each exam. To minimize random error associated with measurement differences and the effect of height loss after age 60, the mean of all measures of adult height up to age 60 years was used in combination with exam-specific weight measures to calculate exam-specific BMI (kg/m^2^). Physical activity was measured by self-report of the number of hours spent each day in sleeping, sedentary, light, moderate, and vigorous activity. A physical activity index was calculated as a weighted average of the total hours of moderate and vigorous activity per day. This index and its associated activity weights were derived from the previous work of Kannel in the Framingham cohort [32]. Cigarette smoking was assessed at every exam and participants were asked about current smoking status, change in smoking status since the last exam, and amount smoked (for current smokers). For these analyses, models controlled for current smoking, which was defined as smoking at least one cigarette per day. Plausible dietary intakes were calculated as a ratio of reported energy intake to the estimated energy requirement. 

### 2.5. Statistical Analyses

The cutoff values for the categories of egg intake were selected by using sensitivity analyses to capture higher and lower levels of intake while optimizing analytical power. The final selected egg consumption categories were as follows: <0.5 eggs, 0.5–<5 eggs, and ≥5 eggs per week. For the analyses of eggs in combination with intakes of other foods, egg consumption and other food intakes were dichotomized using the following cutoff values: eggs, <2.5 vs. ≥2.5 eggs per week; total dairy, <1.75 vs. ≥1.75 cup-equivalents per day; fish, <7 vs. ≥7 ounces per week; whole grains, <0.5 vs. ≥0.5 ounce-equivalents per day; fiber, <15 vs. ≥15 g per day; fruits and non-starchy vegetables, <3 vs. ≥3 cup-equivalents per day. 

An analysis of covariance (ANCOVA) was used to calculate adjusted mean fasting glucose and blood pressure levels after four years of follow-up according to categories of baseline egg consumption. Similarly, adjusted mean levels of fasting glucose and blood pressure were estimated for categories of egg intake combined with healthy eating patterns. For these analyses, BMI was considered to be both an effect modifying variable and a potential causal intermediate. 

Cox proportional hazard models were used to estimate the hazard ratios (HR) and 95% confidence intervals (CI) for incident IFG or T2D (IFG/T2D) and HBP over approximately ten years of follow-up according to egg intake at baseline. Follow-up for incident IFG/T2D and incident HBP started at the time of baseline egg assessment and continued until the first of the following events: incident IFG/T2D (for IFG/T2D analyses) or incident HBP (for HBP analyses), loss to follow-up, end of follow-up, or death. In addition, dichotomous egg consumption was cross-classified with intakes of other foods, including dairy, fish, whole grains, dietary fiber, and fruit and non-starchy vegetables, in order to determine the effects of egg consumption alone and in combination with other foods on the outcomes of interest. Finally, sensitivity analyses were carried out including only those subjects with plausible dietary intakes between 75% and 125% of estimated energy requirements. All analyses were performed using Statistical Analysis Systems software, version 9.4 (SAS Institute, Cary, NC, USA).

## 3. Results

Table 1 shows the baseline characteristics of participants according to their usual egg intake per week. Since individuals who consumed ≥5 eggs per week were more likely to be male, the remaining baseline factors were adjusted for sex. Those who consumed ≥5 eggs per week also had a slightly higher BMI but a somewhat lower fasting glucose level at baseline. They also tended to have lower intakes of fruits and vegetables, lower energy-adjusted intakes of protein, carbohydrates, and SoFAAs, and higher intakes of fats and saturated fats. Finally, higher egg intake was associated with substantially higher intakes of dietary cholesterol.

Table 2 shows that consuming five or more eggs per week was associated with a fasting glucose concentration at follow-up that was 3.7 mg/dL lower than that of subjects consuming less than 0.5 eggs per week (*p* = 0.0004) after adjusting for age, sex, dietary fiber, and BMI. These effects were stronger among overweight (4.5 mg/dL difference) than normal weight individuals (1.6 mg/dL difference). While males generally had higher fasting glucose levels, higher egg intakes were associated with lower glucose levels in both males and females. Consuming five or more eggs per week was also associated with an SBP level that was 2.5 mg/dL lower than that of participants consuming <0.5 eggs/week. This association was strongest in males. In addition, higher egg intakes were inversely associated with both SBP and DBP in overweight individuals.

The effects of eggs as part of eating patterns on mean fasting glucose and blood pressure levels are shown in Table 3. In these analyses, the beneficial effects of eggs on fasting blood glucose were strengthened when consumed in combination with higher intakes of dairy, fish, dietary fiber, and fruits and non-starchy vegetables. For example, those with higher intakes of both eggs and dairy products had a fasting glucose level that was 2.3 mg/dL lower (*p* = 0.0203) than that observed among those with lower intakes in both food groups. Participants with higher egg intakes combined with higher dietary fiber intakes also had lower SBP (*p* = 0.0195) and DBP (*p* = 0.0201) levels compared with those in the referent group. Higher egg and fiber intake alone were associated with lower fasting glucose levels compared with the referent group.

The occurrence of IFG/T2D and HBP according to categories of egg intake is shown in Table 4. Participants consuming five or more eggs per week (vs. <0.5 eggs/week) had a non-statistically significant 28% lower risk of incident IFG/T2D (HR: 0.72; 95% CI: 0.51–1.03) during the follow-up period. These participants with the highest egg intakes also had a 32% lower risk (HR: 0.68; 95% CI: 0.50–0.93) of HBP compared with the referent group (<0.5 eggs/week). These latter results were stronger among males than females

Table 5 shows the effects of egg-related eating patterns on the risk of incident IFG/T2D and HBP. Eating patterns that included eggs generally resulted in a lower incidence of IFG/T2D. In particular, eating patterns that included eggs as well as higher amounts of fiber, fish, and whole grains resulted in a statistically significant 26–29% reduction in the risk of IFG/T2D. In addition, patterns that included higher intakes of eggs in combination with more dairy, fish, fiber, fruits and non-starchy vegetables resulted in a 25–41% lower risk of developing HBP.

## 4. Discussion

In the current study, consuming five or more eggs per week had no adverse effect on fasting glucose over four years of follow-up among healthy adults. These analyses show that higher egg intakes were associated with slightly lower levels of fasting glucose. After stratifying by baseline BMI, overweight individuals benefitted more from egg consumption. Overall, participants with normal fasting glucose at baseline who consumed more eggs had a lower risk of developing IFG or T2D over the next decade. These effects were even stronger in combination with other healthy eating patterns.

Consuming five or more eggs per week was also associated with lower SBP levels, particularly among those who were overweight. The beneficial association between egg consumption and blood pressure was generally stronger among males, and among those with higher intakes of other healthy foods and nutrients. Furthermore, those who consumed five or more eggs per week had about a 30% lower risk of HBP. 

The current results add support to a previous short-term clinical trial finding that egg consumption as part of a high-protein diet led to greater reductions in blood pressure and 2-hour glucose than a comparable diet without eggs [15]. Some other studies found no adverse or beneficial effects of daily egg intake on blood pressure [19,33]. The authors of one of these studies, however, concluded that the null results may have been due to uncontrolled confounding by other dietary and lifestyle factors, particularly in secondary analyses of earlier clinical trials [19].

Concerns about egg consumption and diabetes have existed for a long time, although the results of previous studies of eggs and glucose-related outcomes have been inconsistent. Two meta-analyses found that egg consumption in U.S. studies was associated with a higher risk of T2D, while those from non-U.S. studies found no such association [10,11]. These results may support the idea that differences in other diet or lifestyle factors or methodologic differences between studies may explain some of the differences between studies, which range from the null results cited above to other studies which found that higher egg intakes were associated with lower fasting glucose levels or lower risks of T2D [34,35]. In particular, the variable dietary patterns that accompany egg intake may be responsible for observed differences in the associations between eggs and cardiometabolic health-related outcomes across studies [36].

Several short-term randomized clinical trials found that consuming two to three eggs per day for 12 weeks as part of an energy-restricted diet [13,14,15] had no adverse effects on blood glucose in either females or males. One of these studies among individuals with prevalent T2D found improvements in hemoglobin A1c and fasting glucose associated with consuming two eggs per day as part of a 12-week energy-restricted diet [15]. Several studies further support the idea that higher egg intakes in the setting of generally healthier dietary patterns has no adverse effects on cardiometabolic health and may have beneficial effects. A recent analysis from the ATTICA study in Greece found eggs to be unassociated with type 2 diabetes risk and inversely associated with the risk of both CVD and hypertension [33]. In addition, a study in Taiwan found that vegetarians who ate eggs (ovo-vegetarians) had lower SBP and DBP levels than either vegans or omnivores [37].

In some cultures, eggs have been linked with less healthy eating patterns (e.g., more red meat and fewer whole grains or fruits and vegetables), and this could result in egg consumers having lower intakes of beneficial nutrients such as dietary fiber, B vitamins, or antioxidant vitamins [38]. Thus, it is difficult in some of these studies to separate the effects of eggs from their associated eating patterns [18], and difficult to ascertain whether any increased health risk might be due to egg consumption itself or to lower intakes of other important foods and nutrients. In our analysis, we evaluated egg consumption in combination with other eating patterns. These analyses were made possible through the detailed dietary record data from this study. We found that the beneficial effects of eggs on fasting glucose and blood pressure were often stronger when eggs were combined with higher intakes of other healthy foods and nutrients such as dairy, fish, fruits and non-starchy vegetables, whole grains, and dietary fiber.

The nutrient composition of eggs could explain their beneficial effects on glucose-related outcomes. For instance, the protein content of eggs may play a role in glucose metabolism by serving as a substitute for carbohydrates, which have a higher glycemic load, as a substrate for gluconeogenesis, or by promoting insulin secretion from pancreatic β–cells [39]. Furthermore, egg yolks are a rich source of the carotenoids lutein and zeaxanthin [40] that have been associated with lower 2-hour post-load glucose as well as fasting insulin [41]. These nutrients may play an important role in modulating inflammation via the inhibition of nuclear factor-kappa B, or limiting oxidative stress via interaction with the nuclear factor-erythroid 2-related factor 2 pathway [42]. Finally, eggs are one of a few food sources of vitamin D [43], which may play an important role in glucose metabolism by improving pancreatic β-cell function through both direct and indirect effects on insulin secretion, improvement in insulin action, and the reduction of systemic inflammation [44].

The favorable effects of eggs on blood pressure may be attributed to a number of egg-derived bioactive peptides that have substantial anti-oxidant capacity [45] and may inhibit angiotensin-converting enzymes, thereby lowering blood pressure [46]. In previous mouse studies, investigators demonstrated that egg consumption (especially phospholipid from egg yolk) decreases oxidative stress, and as a result may lower the long-term risk of hypertension [47,48]. In addition, the arginine content in eggs may lower blood pressure by acting as a substrate for nitric oxide synthesis and induce vasodilation [49]. A number of possible mechanisms could also explain the synergistic effects of eggs as a part of a healthy dietary pattern on these cardiometabolic outcomes.

This study has several important strengths. The three-day records provided detailed estimates of individual food intake collected in a standardized fashion. The use of food records instead of a food frequency questionnaire would have enabled a more accurate assessment of egg intake. Furthermore, the wealth of dietary data also enabled us to examine the independent effects of egg intake on cardiovascular risk factors as well as the effects of eggs combined with other dietary factors. Finally, a number of potential confounders were systematically collected in the Framingham Study, thus enhancing the validity of the results. 

This study also has several limitations. The dietary data is self-reported and potentially subject to random error and reporting bias. In addition, out of the 5124 participants enrolled in FOS, only 3284 (64%) provided dietary records. Furthermore, these dietary records were only collected between exams three and five, and were not available during follow-up exams. Finally, the range of egg intake was limited, which could be the result of individuals following the diet policy at the time to reduce their egg intake. 

## 5. Conclusions

This prospective study suggests that consuming five or more eggs per week does not adversely affect glucose or blood pressure-related outcomes. In fact, moderate intakes of eggs may have beneficial effects on blood glucose and the long-term risk of IFG and T2D. Furthermore, the moderate intake of eggs were linked with lower systolic blood pressure and a significantly lower risk of developing incident HBP. Overall, these results provide no evidence to restrict egg intake to reduce the risk of elevated glucose or HBP in healthy adults. Rather, moderate amounts of eggs may reduce the risk of impaired fasting glucose, type 2 diabetes, or high blood pressure when consumed as part of a healthy eating pattern.

## Figures and Tables

**Table 1:** Baseline characteristics of subjects in the Framingham Offspring Study according to egg consumption ^1^.

Weekly Number of Eggs Consumed				
	0 to 0.5	0.5 to <5	≥5	*p*-Trend
Subjects, *n*	353	1329	372	
Male, *n* (%)	133 (37.7%)	568 (42.7%)	223 (60.0%)	<0.0001
Smoker, *n* (% current)	79 (22.4%)	285 (21.4%)	98 (26.3%)	0.14
More than high school ^2^, *n* (%)	178 (57.2%)	754 (63.2%)	201 (61.1%)	0.15
Age, years	49.6 ± 0.47	48.6 ± 0.24	48.1 ± 0.46	0.07
Height, cm	168.5 ± 0.33	168.5 ± 0.18	169.2 ± 0.33	0.18
BMI, kg/m^2^	26.2 ± 0.23	26.0 ± 0.12	26.8 ± 0.23	0.003
Systolic blood pressure, mmHg	124.1 ± 0.84	122.4 ± 0.43	122.1 ± 0.82	0.15
Diastolic blood pressure, mmHg	78.4 ± 0.49	77.8 ± 0.25	77.8 ± 0.48	0.53
Fasting glucose, mg/dL	95.4 ± 0.71	92.5 ± 0.36	92.7 ± 0.69	0.001
Physical activity index	12.7 ± 0.42	12.4 ± 0.22	13.0 ± 0.41	0.30
Energy intake, kcals/day	1722 ± 24.1	1919 ± 12.4	2074 ± 23.6	<0.0001
Dietary cholesterol, mg/day	173 ± 4.19	242 ± 2.16	402 ± 4.11	<0.0001
Protein, % of energy	17.7 ± 0.17	16.8 ± 0.09	16.4 ± 0.17	<0.0001
Carbohydrate, % of energy	47.4 ± 0.42	46.2 ± 0.22	43.9 ± 0.41	<0.0001
Fat, % of energy	32.9 ± 0.34	35.1 ± 0.18	38.0 ± 0.33	<0.0001
Saturated fat, % of energy	10.9 ± 0.15	12.0 ± 0.08	13.3 ± 0.15	<0.0001
SoFAAs, % energy	12.5 ± 0.28	10.3 ± 0.14	8.7 ± 0.27	<0.0001
FNSV, cup equivalents/day	2.6 ± 0.07	2.6 ± 0.04	2.3 ± 0.07	0.006
Whole grains, ounce equivalents/day	0.6 ± 0.04	0.6 ± 0.02	0.5 ± 0.04	0.17
Dairy, cup equivalents/day	1.3 ± 0.05	1.4 ± 0.02	1.4 ± 0.05	0.16
Dietary fiber, grams/day	16.1 ± 0.32	16.1 ± 0.16	15.5 ± 0.31	0.16

^1^ Data were adjusted means ± standard error, unless otherwise noted. All means were adjusted for sex. ^2^ Subjects missing education data were included in the analysis by use of a dummy variable. Abbreviations: Fruit and non-starchy vegetables (FNSV), Solid fats, alcoholic beverages, and added sugars (SoFAAs).

**Table 2:** Effects of egg intake on fasting glucose and blood pressure after four years of follow-up, stratifying by baseline BMI and by sex ^1^.

	All Subjects		BMI < 25 kg/m^2^		BMI ≥ 25 kg/m^2^		Females		Males	
Egg Intake/Week	*n*	Mean ± SE	*n*	Mean ± SE	*n*	Mean ± SE	*n*	Mean ± SE	*n*	Mean ± SE
Fasting glucose (mg/dL)										
<0.5	353	96.6 ± 0.73	157	90.9 ± 0.72	196	101.0 ± 1.20	220	94.2 ± 0.90	133	99.5 ± 1.21
0.5 to <5	1329	93.2 ± 0.38	610	88.7 ± 0.37	719	96.4 ± 0.63	761	90.6 ± 0.49	568	96.4 ± 0.59
≥5	372	92.9 ± 0.72	132	89.3 ± 0.79	240	96.5 ± 1.09	149	90.4 ± 1.10	223	96.0 ± 0.94
*p*-trend		0.0004		0.0979		0.0098		0.0025		0.0450
Systolic blood pressure (mmHg)										
<0.5	354	125.7 ± 0.78	164	119.4 ± 1.13	190	131.1 ± 1.09	223	122.7 ± 1.01	131	129.9 ± 1.20
0.5 to <5	1269	123.6 ± 0.41	618	118.6 ± 0.58	651	127.8 ± 0.59	743	121.6 ± 0.55	526	126.1 ± 0.60
≥5	374	123.3 ± 0.76	143	120.0 ± 1.21	231	127.0 ± 0.99	147	121.3 ± 1.25	227	125.8 ± 0.91
*p*-trend		0.0284		0.7581		0.0071		0.3419		0.0173
Diastolic blood pressure (mmHg)										
<0.5	354	78.6 ± 0.47	164	74.3 ± 0.69	190	82.3 ± 0.65	223	75.8 ± 0.61	131	82.4 ± 0.75
0.5 to <5	1269	77.6 ± 0.25	618	74.5 ± 0.35	651	80.1 ± 0.35	743	75.4 ± 0.33	526	80.3 ± 0.37
≥5	374	77.6 ± 0.46	143	75.0 ± 0.74	231	80.2 ± 0.60	147	74.9 ± 0.75	227	80.7 ± 0.57
*p*-trend		0.1157		0.5064		0.0279		0.3783		0.1530

^1^ Models (except sex-specific models) were adjusted for age, sex, dietary fiber, and BMI. BMI stratified means were adjusted for age, sex, and dietary fiber. Abbreviations: Standard error (SE).

**Table 3:** Effects of egg intake as part of healthy patterns on mean fasting glucose and blood pressure levels after four years of follow-up ^1^.

	Fasting Glucose (mg/dL)			SBP (mmHg)			DBP (mmHg)		
Baseline Diet Pattern ^2^	*n*	Mean ± SE	*p*-Value	*n*	Mean ± SE	*p*-Value	*n*	Mean ± SE	*p*-Value
Eggs/Dairy									
Lower/Lower	876	94.2 ± 0.47	-	841	124.3 ± 0.51	-	841	78.1 ± 0.31	-
Lower/Higher	294	94.1 ± 0.80	0.9154	287	124.5 ± 0.86	0.8574	287	77.2 ± 0.52	0.1427
Higher/Lower	621	93.7 ± 0.55	0.4693	608	123.7 ± 0.59	0.4646	608	77.8 ± 0.36	0.5196
Higher/Higher	263	91.9 ± 0.86	0.0203	261	122.5 ± 0.91	0.0793	261	77.0 ± 0.56	0.0784
Eggs/Fish									
Lower/Lower	592	94.6 ± 0.57	-	582	124.4 ± 0.61	-	582	77.9 ± 0.37	-
Lower/Higher	578	93.7 ± 0.57	0.2221	546	124.3 ± 0.62	0.9680	546	77.9 ± 0.38	0.9755
Higher/Lower	461	94.0 ± 0.64	0.4424	470	123.4 ± 0.67	0.3046	470	77.3 ± 0.41	0.2954
Higher/Higher	423	92.2 ± 0.67	0.0063	399	123.3 ± 0.73	0.2545	399	77.9 ± 0.45	0.9935
Eggs/WG									
Lower/Lower	656	94.7 ± 0.54	-	626	124.9 ± 0.58	-	626	78.2 ± 0.36	-
Lower/Higher	514	93.5 ± 0.61	0.1279	502	123.6 ± 0.65	0.1396	502	77.5 ± 0.40	0.2483
Higher/Lower	516	93.2 ± 0.61	0.0762	501	123.1 ± 0.65	0.0331	501	77.2 ± 0.40	0.0851
Higher/Higher	368	93.0 ± 0.72	0.0591	368	123.8 ± 0.76	0.2377	368	78.0 ± 0.47	0.8443
Eggs/Fiber									
Lower/Lower	765	94.9 ± 0.57	-	737	125.0 ± 0.54	-	737	78.5 ± 0.33	-
Lower/Higher	405	92.9 ± 0.58	0.0136	391	123.1 ± 0.74	0.0364	391	76.8 ± 0.45	0.0018
Higher/Lower	585	93.3 ± 0.67	0.0351	574	123.7 ± 0.61	0.1135	574	77.8 ± 0.37	0.1917
Higher/Higher	299	92.9 ± 0.65	0.0367	295	122.7 ± 0.85	0.0195	295	77.1 ± 0.52	0.0201
Eggs/FNSV									
Lower/Lower	786	94.4 ± 0.49	-	769	124.7 ± 0.53	-	769	78.1 ± 0.32	-
Lower/Higher	384	93.5 ± 0.70	0.2779	359	123.6 ± 0.77	0.2437	359	77.4 ± 0.47	0.2536
Higher/Lower	634	93.4 ± 0.55	0.1610	631	123.5 ± 0.58	0.1360	631	77.6 ± 0.36	0.3121
Higher/Higher	250	92.4 ± 0.87	0.0448	238	122.9 ± 0.95	0.1060	238	77.5 ± 0.58	0.3697

^1^ All means were adjusted for age, sex, and BMI. ^2^ Lower vs. higher intakes: <2.5 vs. ≥2.5 eggs per week; <1.75 vs. ≥1.75 servings of dairy per day; <7 vs. ≥7 ounces of fish per week; <0.5 vs. ≥0.5 ounce-equivalents of whole grains per day; <15 vs. ≥15 g of fiber per day; <3 vs. ≥3 cup equivalents of fruit and non-starchy vegetables per day. Abbreviations: Diastolic blood pressure (DBP), fruit and non-starchy vegetables (FNSV), systolic blood pressure (SBP), whole grains (WG).

**Table 4:** Occurrence of impaired fasting glucose or type 2 diabetes and high blood pressure associated with egg intake, overall, and stratifying by sex.

Egg Intake/Week	*n*	PY	Cases	Incidence Rate/1000 PY	HR (95% CI) ^1^
All Subjects					
IFG/T2D ^2^					
<0.5	316	3364.7	60	17.83	1.00
0.5 to <5	1254	14,318.8	219	15.30	0.74 (0.55, 0.98)
≥5	347	3779.4	74	19.58	0.72 (0.51, 1.03)
High blood pressure					
<0.5	303	3082.6	93	30.17	1.00
0.5 to <5	1142	12,629.0	337	26.69	0.90 (0.71, 1.14)
≥5	323	3511.0	78	22.22	0.68 (0.50, 0.93)
Females					
IFG/T2D					
<0.5	204	2155.6	33	15.31	1.00
0.5 to <5	727	8478.0	81	9.55	0.56 (0.37, 0.84)
≥5	143	1529.7	24	15.69	0.63 (0.36, 1.10)
High blood pressure					
<0.5	199	2096.1	51	24.33	1.00
0.5 to <5	685	7645.0	192	25.12	1.08 (0.79, 1.48)
≥5	135	1500.7	28	18.66	0.68 (0.42, 1.09)
Males					
IFG/T2D					
<0.5	112	1209.2	27	22.33	1.00
0.5 to <5	527	5840.7	138	23.63	0.97 (0.64, 1.48)
≥5	204	2249.7	50	22.23	0.87 (0.54, 1.41)
High blood pressure					
<0.5	104	986.5	42	42.58	1.00
0.5 to <5	457	4984.0	145	29.09	0.71 (0.50, 1.00)
≥5	188	2010.3	50	24.87	0.62 (0.40, 0.94)

^1^ Models for all subjects were adjusted for age, sex, solid fats/alcoholic beverages/added sugars, and BMI. Sex was not included in the models stratifying by sex. ^2^ IFG/T2D is defined as fasting glucose ≥110 mg/dL and/or a T2D diagnosis. Abbreviations: Body mass index (BMI), hazard ratio (HR), impaired fasting glucose (IFG), type 2 diabetes (T2D); person-years (PY).

**Table 5:** Effects of egg-related diet patterns on risks of impaired fasting glucose and high blood pressure.

	IFG/Type 2 Diabetes ^1^		High Blood Pressure ^2^	
Baseline Diet Pattern ^3^	*n*	HR (95% CI)	*n*	HR (95% CI)
Eggs/Dairy				
Lower/Lower	813	1.00	740	1.00
Lower/Higher	274	1.42 (1.04, 1.93)	254	0.92 (0.70, 1.20)
Higher/Lower	579	1.00 (0.78, 1.29)	542	0.81 (0.66, 1.00)
Higher/Higher	251	0.81 (0.57, 1.15)	232	0.75 (0.56, 1.00)
Eggs/Fish				
Lower/Lower	548	1.00	507	1.00
Lower/Higher	539	0.78 (0.59, 1.04)	487	0.89 (0.71, 1.12)
Higher/Lower	431	0.90 (0.67, 1.20)	412	0.86 (0.67, 1.10)
Higher/Higher	399	0.71 (0.52, 0.95)	362	0.73 (0.56, 0.94)
Eggs/WG				
Lower/Lower	604	1.00	552	1.00
Lower/Higher	483	0.93 (0.70, 1.24)	442	0.88 (0.70, 1.11)
Higher/Lower	479	1.01 (0.77, 1.32)	444	0.72 (0.57, 0.92)
Higher/Higher	351	0.71 (0.51, 0.98)	330	0.88 (0.68, 1.13)
Eggs/Fiber				
Lower/Lower	563	1.00	523	1.00
Lower/Higher	524	0.84 (0.63, 1.13)	471	0.71 (0.56, 0.91)
Higher/Lower	393	0.95 (0.70, 1.27)	374	0.86 (0.68, 1.10)
Higher/Higher	437	0.74 (0.55, 1.00)	400	0.59 (0.46, 0.77)
Eggs/ FNSV				
Lower/Lower	733	1.00	680	1.00
Lower/Higher	354	0.73 (0.53, 1.01)	314	0.76 (0.59, 0.99)
Higher/Lower	593	0.84 (0.66, 1.08)	568	0.79 (0.64, 0.98)
Higher/Higher	237	0.77 (0.55, 1.08)	206	0.71 (0.52, 0.97)

^1^ Model for IFG/Type 2 diabetes was adjusted for age, sex, healthy eating index, and BMI. All other IFG/Type 2 diabetes models were adjusted for age, sex, and BMI. IFG/Type 2 diabetes is defined as fasting glucose ≥110 mg/dL and/or a type 2 diabetes diagnosis. ^2^ High blood pressure models were adjusted for age, sex, BMI, and % energy from saturated fat (except for model for Eggs/Dairy). ^3^ Lower vs. higher intakes: <2.5 vs. ≥2.5 eggs per week; <1.75 vs. ≥1.75 servings of dairy per day; <7 vs. ≥7 ounces of fish per week; <0.5 vs. ≥0.5 ounce-equivalents of whole grains per day; <15 vs. ≥15 g of fiber per day; <3 vs. ≥3 cup equivalents of fruit and non-starchy vegetables per day. Abbreviations: Body mass index (BMI), confidence interval (CI), fruit and non-starchy vegetables (FNSV), hazard’s ratio (HR), impaired fasting glucose (IFG), whole grains (WG).

## Data Availability

Data from the Framingham Studies are publicly available through the National Heart, Lung, and Blood Institute’s BIOLINCC data repository (https://biolincc.nhlbi.nih.gov/studies/framoffspring/) (accessed on 17 January 2023). These data were obtained from the Framingham Heart Study and may be requested from (https://www.framinghamheartstudy.org/fhs-for-researchers/data-available-overview/) (accessed on 17 January 2023).
